# Acute and persistent symptoms of COVID-19 infection in school-aged children: a retrospective study from China

**DOI:** 10.1186/s12889-024-17822-5

**Published:** 2024-02-01

**Authors:** Honglin Wang, Fangfang Lu, Xiuxian Ni, Rijing Luo, Linxiang Chen, Jing Yuan, Zhen Zhang, Qiuying Lv

**Affiliations:** 1 Department of Communicable Diseases Control and Prevention, Shenzhen Center for Disease Control and Prevention, 8 Longyuan Rd, 518055 Shenzhen, China; 2 Disease Control Division, Public Health Service Center, Bao’an District, 518103 Shenzhen, China; 3 Department of Epidemiology, School of Public Health, (Guangdong Provincial Key Laboratory of Tropical Disease Research), Southern Medical University, 510515 Guangzhou, China; 4 Department of Immunization Planning, Luohu District Center for Disease Control and Prevention, 518020 Shenzhen, China; 5 Shenzhen Key Laboratory of Pathogen and Immunity, State Key Discipline of Infectious Disease, Shenzhen Third People’s Hospital, Second Hospital, National Clinical Research Center for Infectious Disease, Southern University of Science and Technology, 518112 Shenzhen, China

**Keywords:** COVID-19 infection, School-aged children, Symptoms, SARS-Cov-2, Long COVID

## Abstract

**Background:**

The long-term sequelae of Coronavirus disease 2019 (COVID-19) in children are unclear. We investigated COVID-19 symptoms in school-aged children to determine their impact on patients and their families.

**Methods:**

This cross-sectional study, conducted on February 25–28, 2023, selected a representative kindergarten and 9-year school from Shenzhen, China. There were randomly two classes each for the 12 grades from kindergarten to junior high school. The school-aged children were aged 3–16 years. Literate parents completed an online questionnaire related to their children’s COVID-19 symptoms since December 1, 2022. Descriptive statistics were computed as necessary. Univariate and multivariable linear regression analyses were performed, and variables with a p-value < 0.05 were considered to have a significant association with the subjective feeling scores for COVID-19 infection.

**Results:**

We included 936 school-aged children, with a COVID-19 infection rate of 68.5%. The prevalence of LC 28 (illness with symptoms lasting for 28 days) was 3.4%. During acute infection, the median number of the 641 children’s symptoms was 3.0 (IQR: 1.0–5.0) and the median score of subjective feelings was 15.0 (IQR: 11.0–24.5). The top three symptoms were fever, cough/expectoration, and rhinobyon. Age of 13–16 years (adjusted beta: 3.60, 95% CI: 0.32–6.88) and comorbidities (adjusted beta: 3.47, 95% CI: 1.20–5.73) were independently associated with higher subjective feelings (*p* < 0.05). The top three characteristics associated with LC 28 were alopecia (33.3%, 5/15), cognitive dysfunction (29.2%, 7/24), and emotional problem (28.6%, 6/21).

**Conclusions:**

Children with COVID-19 have a short duration of symptoms and milder symptoms, so they can self-medicate to minimize hospital crowding. Children with basic diseases require prompt attention. Although LC 28 is uncommon in children, mental and psychological problems after COVID-19 recovery should not be ignored.

## Introduction

Severe acute respiratory syndrome coronavirus 2 (SARS-CoV-2) was declared a pandemic 3 years ago on March 11, 2020 by the World Health Organization [1]. As of April 9, 2023, over 762 million confirmed cases and over 6.8 million deaths have been reported globally [2]. At present, Omicron and its sublineages are the most common variants. Approximately 3.8 billion people or 46% of the global population are estimated to have been infected by the Omicron variant and its sublineages [3].

Some adults with Coronavirus disease 2019 (COVID-19) experience prolonged illness duration, known as “long COVID” [4, 5]. Longitudinal data from the King’s College London COVID Symptom Study [6] showed that 13.3% of adults with a positive test for SARS-CoV-2 had symptoms for at least 4 weeks (referred to as LC28) and 4.5% had symptoms for at least 8 weeks (LC56) [7]. However, these data were obtained when previous variants were in circulation. Thus, one important issue is the risk of long COVID after infection with the Omicron variant. The potential implications are enormous as Omicron has infected an unprecedented number of people worldwide. If many of the infected patients develop long COVID, millions could be burdened with debilitating symptoms for months.

Moreover, most post-COVID studies have focused on adults [8], given the predominance of adult COVID-19 in the first pandemic waves, which appeared to spare children to a certain extent. Therefore, a limited number of studies have been conducted in children [9], although the need for research on COVID-19 sequelae in children and young individuals has been previously acknowledged and grown in importance with the emergence of variants that affect children [10].

With the end of the emergence of new outbreaks by the SARS-CoV-2 Omicron lineages BA5.2 and BF.7 in China since December 2022, there is a need to assess the long-term consequences of COVID-19 in pediatric populations [11]. Thus, we investigated the acute and persistent symptoms of COVID-19 infection in school-aged children to inform clinicians, researchers, and public health experts regarding the impacts of this condition on patients and their families, as well as to inform discussions regarding medical resource allocation.

## Methods

### Study design and participants

A cross-sectional study was conducted in China from February 25 to February 28, 2023, to assess the acute and persistent symptoms of COVID-19 infection in school-aged children. The ethical review committee of Shenzhen Center for Disease Control and Prevention (ERC Number: SZCDCLL-[2022]010A) approved this study. An anonymous online questionnaire in the official languages of China was developed. The questionnaire was designed to be filled by parents of students enrolled in kindergarten, primary, or junior high schools. The questionnaire included demographics, basic diseases, parental education level, COVID-19 infections, acute symptoms and duration, subjective feelings, treatment, and persistent symptoms. Informed consent was obtained from all study participants before the study procedures.

The sample size was determined using a single population proportion formula considering the following assumptions: 95% confidence level (1.96), 4.4% [19] of LC28 was considered, 1.3% margin of error, and 10% nonresponse rate. Accordingly, the final sample size becomes 916.

### Procedures

A multistage sampling technique was employed to select the study participants. First, we selected a kindergarten and a 9-year school in Shenzhen, China, which had a high degree cooperation with us. In addition, these two institutions were large and had sufficiently representative school-aged children sample sizes to meet our sampling needs. Second, there were 12 grades from kindergarten to junior high school. We selected randomly two random classes from each grade, with school-aged children aged 3–16 years. The online questionnaire was distributed to an online communication group comprising of school doctors and teachers. Following parental consent, literate parents completed the questionnaire based on their observations and communications regarding their children’s acute and persistent symptoms of COVID-19 infection since December 1, 2022.

COVID-19 positivity was defined as a positive antigen (AG) or nucleic acid (polymerase chain reaction [PCR]) test, or the presence one of the 10 symptoms of COVID-19. The top 10 acute symptoms of COVID-19 included fever, diarrhea, rhinobyon, runny nose, myalgias, conjunctivitis, sore throat, fatigue, cough/expectoration, and hyposmia/hypogeusia. We also designed a scale to assess children’s subjective feelings of the 10 symptoms, with parents scoring each symptom on a scale of 1–10, with 1 being the least severe and 10 being the most severe. The total score for the scale was 100.

Persistent symptoms were calculated as the number of symptoms reported at least once over defined timeframes (during the first week, first 28 days, from day 28 until illness end, and the entire illness duration) [7]. Illness with symptoms lasting for 28 days or more and for 56 days or more were termed LC 28 and LC 56, respectively.

### Statistical analysis

Data were presented using descriptive statistics. Continuous variables were summarized as median (interquartile range, IQR) and categorical variables as frequency (percentage). Percentile bars were used to show the distribution of symptoms and duration in acute and persistent COVID-19 infection. Heat maps were used to describe the subjective feelings of each symptom in the acute phase. We performed univariable and multivariable linear regression analyses to examine the relation between gender, age, vaccinations, basic disease and the subjective feeling scores for COVID-19 infection. To identify characteristics that were independently associated with subjective feeling scores, we applied backward selection. Variables selected as potential correlates were based on previous research on correlates of COVID-19 infection. All variables were entered in the model and subsequently removed one by one, starting with the variable with the highest ***P***-value, until only signifcant (***P*** < 0.05) correlates remained. The 95% confidence intervals and beta coefficients were calculated and used to describe the statistically significant covariates. ***P***-value < 0.05 was considered statistically significant. We performed the analyses using R version 4.2.1 and IBM SPSS version 26.

## Results

### Population description and COVID-19 infection

**Fig. 1:**
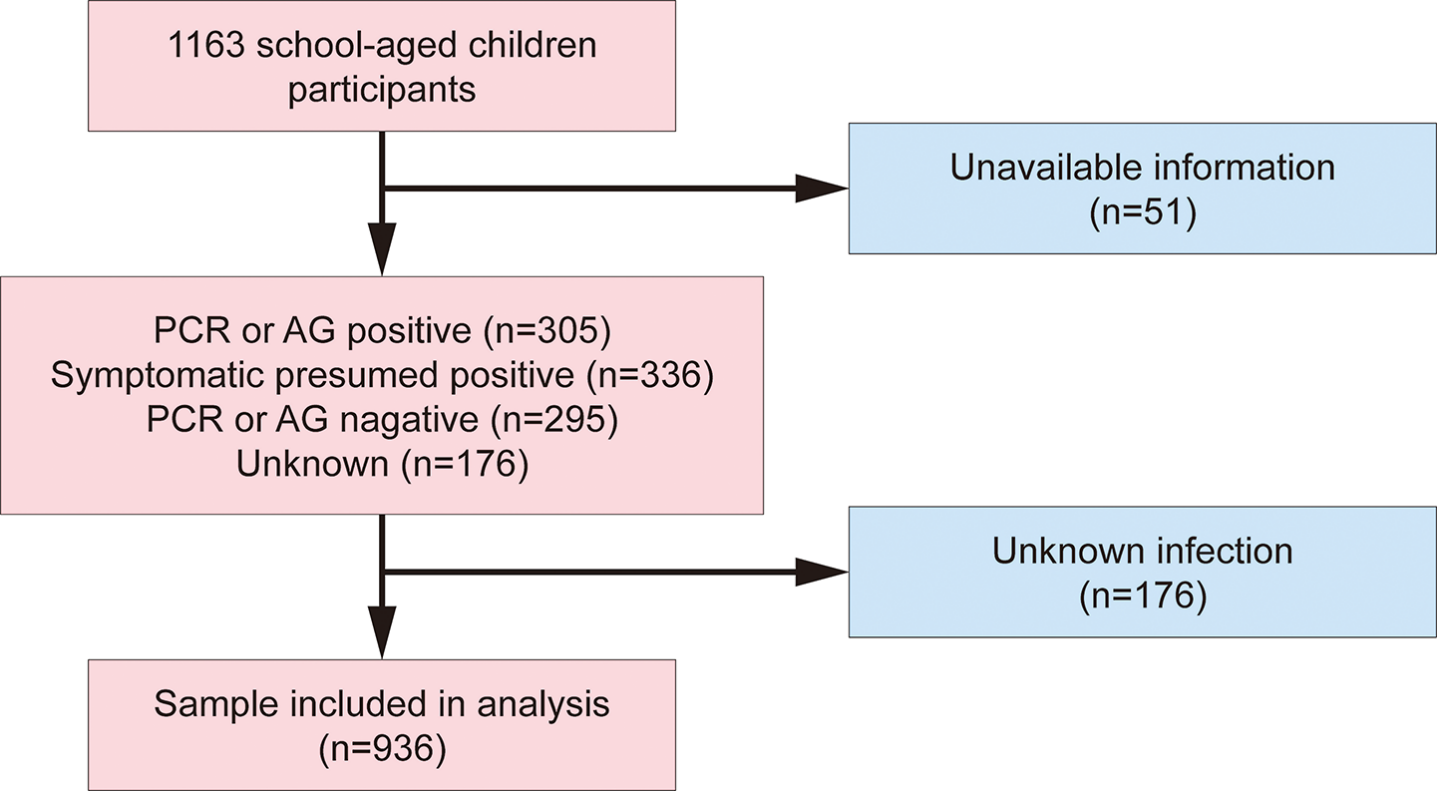
Flow diagram of participant selection

As outlined in Figs. 1, 1163 parents who responded to the online survey. Of the 51 parents who did not complete the questionnaire completely, 176 were unaware of their children’s COVID-19 status. Thus, the final sample size was 936 (participation rate: 80.5%, meeting our sample size requirements), with 503 (53.7%) males and 433 (46.3%) females. The median (interquartile range [IQR]) age was 10.0 (IQR: 8.0–12.0) years (range 3–16 years). The majority of children had highly educated parents (junior college or above, 67.6%), no basic diseases (77.8%), and received two COVID-19 vaccines (91.5%).

In total, 641 children had a COVID-19 infection after December 1, 2022, with a positivity rate of 68.5%. The median time between their last vaccination and infection was 12.6 (IQR: 11.6–13.1) months, with the majority (68.8%) having been vaccinated for longer than 12 months. The median number of symptoms during the acute infection was 3.0 (IQR: 1.0–5.0), with most (46.2%) having 1–3 symptoms. Moreover, 67.7% of children felt that their symptoms were mild, with a subjective feeling score < 20. Most children (72.7%) were treated by taking medications at home. The characteristics of the study population are listed in Table 1.

**Table 1:** Socio–demographic and COVID-19 infection participants of survey participants (*n* = 936)

Characteristic	Frequency	%
**Gender**		
Male	503	53.7
Female	433	46.3
**Age (years)**		
3–6	185	19.8
7–9	237	25.3
10–12	296	31.6
13–16	218	23.3
**Parental education level**		
Junior high school or below	141	15.1
Senior high school	162	17.3
Junior college	343	36.6
College or above	290	31.0
**Presence of basic diseases** ^*^		
No	728	77.8
Yes	208	22.2
**Vaccination doses**		
None/one	80	8.5
Two	856	91.5
**COVID-19 infection**		
Negative	295	31.5
Positive	641	68.5
**Time between last vaccination and COVID-19 infection (** ***n*** ** = 574)** ^**#**^		
< 6 Months	47	8.2
6–12 Months	132	23.0
> 12 Months	395	68.8
**Number of symptoms (** ***n*** ** = 641)**		
0	75	11.7
1–3	296	46.2
> 3	270	42.1
**Subjective feeling score (** ***n*** ** = 641)**		
< 20	434	67.7
20–40	160	25.0
> 40	47	7.3
**Treatment (** ***n*** ** = 641)**		
Self-medication at home	466	72.7
Required medical attention	29	4.5
Both self-medication and required medical attention	34	5.3
Did not take medication	112	17.5

^*^Basic diseases included genetic disorders (9, 1.0%), birth defects (1, 0.1%), hypertension (1, 0.1%), pneumonia (4, 0.4%), asthma (5, 0.5%), allergic rhinitis (138, 14.7%), eczema (27, 2.9%), food allergies (26, 2.8%), and history of surgery (25, 2.7%). ^#^Missing data

### Acute symptoms and duration

**Fig. 2:**
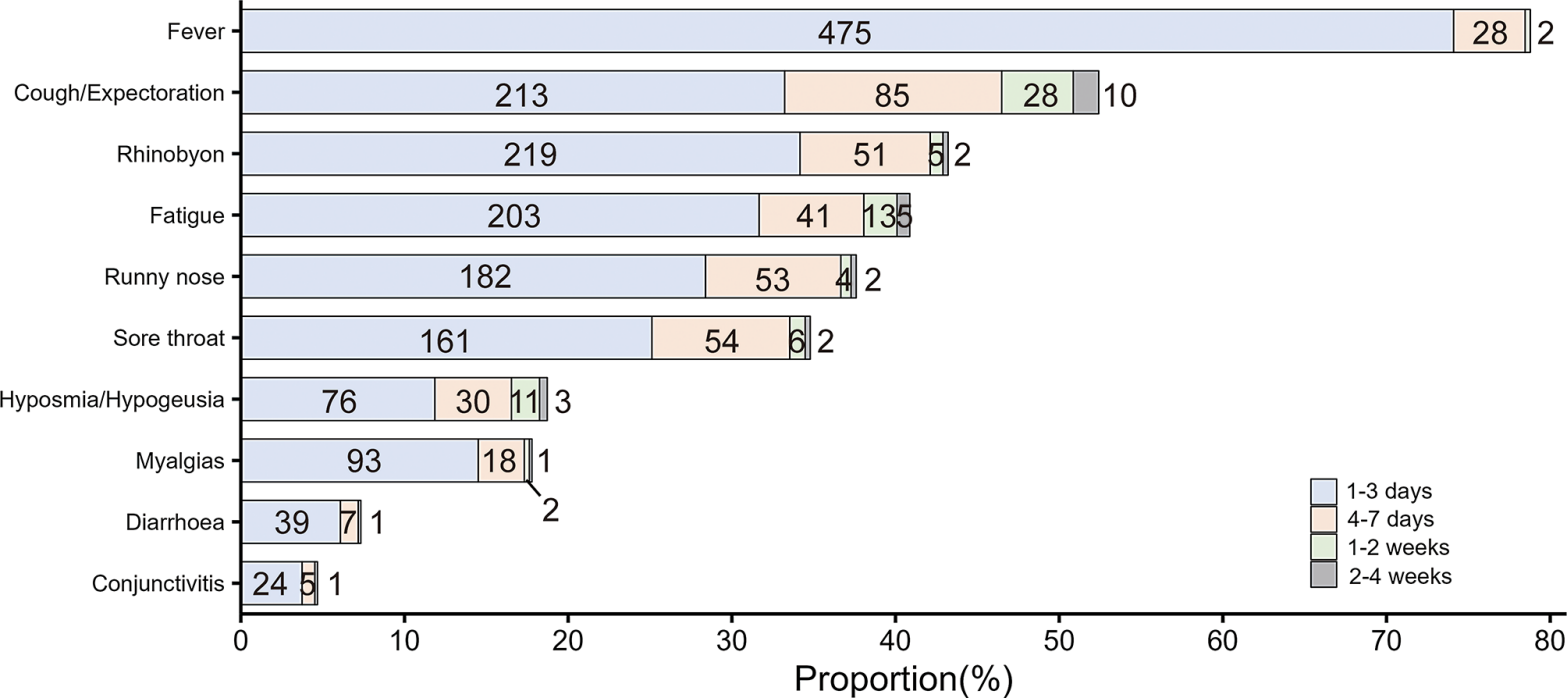
Acute symptoms and their duration in school-aged children with COVID-19 infection

Among the 641 COVID-19-positive children, 88.3% (566/641) had symptoms and 11.7% (75/641) were asymptomatic. The median number of symptoms during acute infection was 3.0 (IQR: 1.0–5.0). The top three symptoms were fever (78.8%, 505/641), cough/expectoration (52.4%, 336/641), and rhinobyon (43.2%, 277/641), which typically lasted for 1–3 days (94.1% [475/505], 63.4% [213/336], 79.1% [219/277]), whereas some lasted for 4–7 days (5.5% [28/505], 25.3% [85/336], 18.4% [51/277]) and a few lasted longer than 7 days (0.4% [2/505], 11.3% [38/336], 2.5% [7/277]). Among the 10 symptoms, the proportion of symptoms that lasted for less than 7 days was as follows, in the descending order: fever (99.6%, 503/505), diarrhea (97.9%, 46/47), rhinobyon (97.5%, 270/277), runny nose (97.5%, 235/241), myalgias (97.4%, 111/114), conjunctivitis (96.7, 29/30), sore throat (96.4%, 215/223), fatigue (93.1%, 244/262), cough/expectoration (88.7%, 298/336), and hyposmia/hypogeusia (88.3%, 106/120). The detailed results are shown in Fig. 2.

### Subjective feelings and associated factors of acute symptoms

**Fig. 3:**
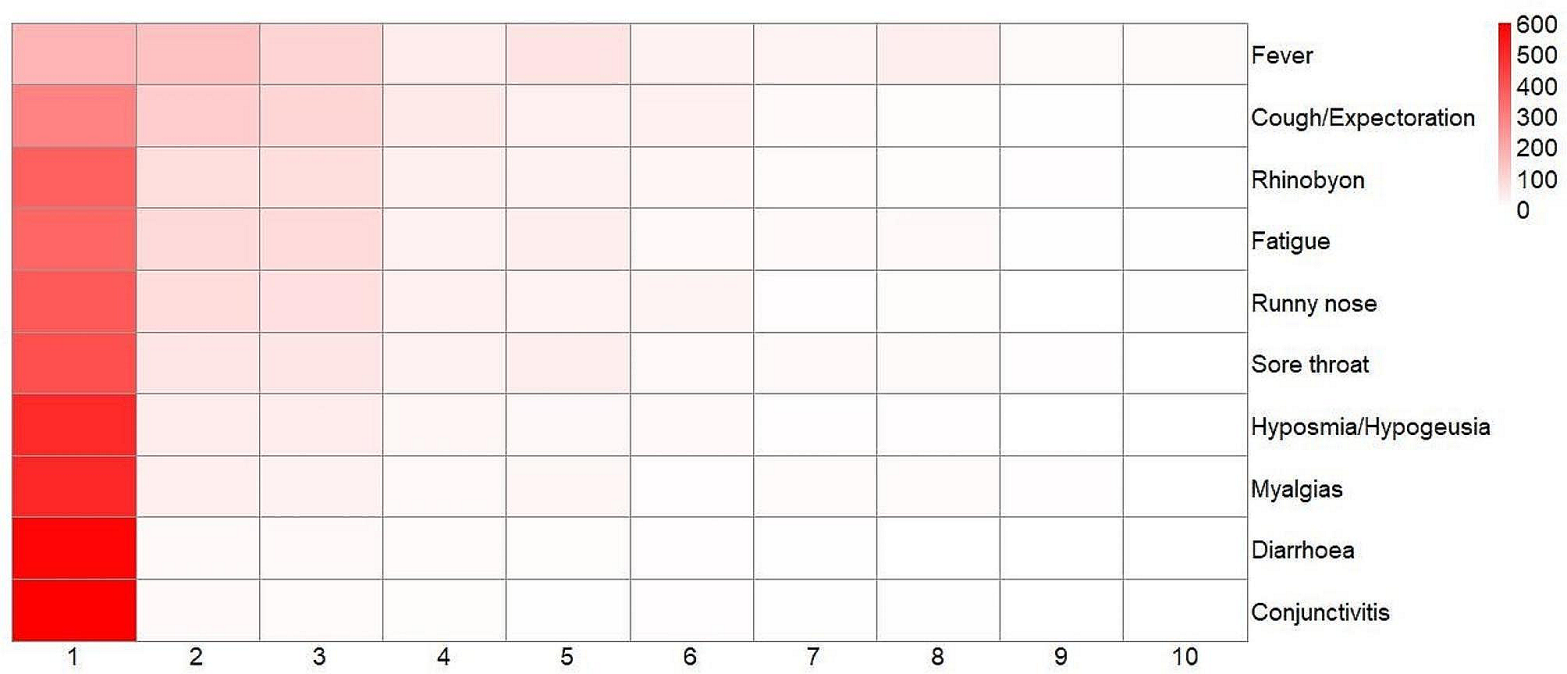
Subjective feelings of acute symptoms in school-aged children with COVID-19 infection

The median score of the 641 children’s subjective feelings was 15.0 (IQR: 11.0–24.5). The top three most severe symptoms were fever (3.0, IQR: 1.0–5.0), cough/expectoration (2.0, IQR: 1.0–3.0), and rhinobyon (1.0, IQR: 1.0–3.0). Conjunctivitis (1.0, IQR: 1.0–1.0), diarrhea (1.0, IQR: 1.0–1.0), and myalgias (1.0, IQR: 1.0–1.0) were associated with lower subjective feeling scores. Figure 3 presents the acute symptom scores of children’s subjective feelings.

Univariate linear regression model analyses revealed that significantly increases in subjective feeling scores were found in participants who had age of 10–12 years (unadjusted beta: 2.87, 95% confidence interval [CI]: 0.08–5.67), or age of 13–16 years (unadjusted beta: 3.37, 95% CI: 0.36–6.38), vs. age of 3–6 years, participants with basic diseases (unadjusted beta: 2.69, 95% CI: 0.50–4.88) vs. participants without basic diseases. Gender and vaccinations were not signifcantly associated with the subjective feeling scores.

Multivariable linear regression model analyses revealed that significantly increases in subjective feeling scores were found in participants who had age of 13–16 years (adjusted beta: 3.60, 95% CI: 0.32–6.88) vs. age of 3–6 years, participants with basic diseases (adjusted beta: 3.47, 95% CI: 1.20–5.73) vs. participants without basic diseases. Gender and vaccinations were also not signifcantly associated with the subjective feeling scores. The detailed results are shown in Table 2.

**Table 2:** Linear regression analysis for subjective feelings and associated factors of acute symptoms in school-aged children with COVID-19 infection

Associated factors	Positive SARS- CoV- 2 (*n* = 641 [%])	Unadjusted				Adjusted		
		Beta (95% CI)		***p-value***		Beta (95% CI)		***p-value***
**Gender**								
Male	351 (69.8)	Reference				Reference		
Female	290 (67.0)	1.36 (-0.53–3.25)		0.158		1.19 (-0.74–3.12)		0.227
**Age (years)**								
3–6	108 (58.4)	Reference				Reference		
7–9	175 (73.8)	0.09 (-2.81–2.99)		0.952		-0.36 (-3.53–2.80)		0.821
10–12	213 (72.0)	2.87 (0.08–5.67)		0.044^*^		2.94 (-0.16–6.04)		0.064
13–16	145 (66.5)	3.37 (0.36–6.38)		0.028^*^		3.60 (0.32–6.88)		0.032^*^
**Time between last vaccination and COVID-19 infection (** ***n*** ** = 574)** ^**#**^ **)**								
<6 Months	47 (100.0)	Reference				Reference		
6–12 Months	132 (100.0)	0.52 (-3.40–4.43)		0.795		0.94 (-2.93–4.92)		0.619
>12 Months	395 (100.0)	1.14 (-2.41–4.70)		0.528		1.24 (-2.37–4.85)		0.677
**Presence of basic diseases** ^*^								
No	487 (66.9)	Reference				Reference		
Yes	154 (74.0)	2.69 (0.50–4.88)		0.016^*^		3.47 (1.20–5.73)		0.003^*^

^*^p < 0.05. CI, confidence interval; basic diseases included genetic disorders (8, 88.9%), birth defects (1, 100.0%), pneumonia (4, 100.0%), asthma (3, 60.0%), allergic rhinitis (103, 74.6%), eczema (18, 66.7%), food allergies (21, 80.8%), and history of surgery (19, 76.0%). ^#^Missing data

### Prevalence and duration of LC 28

In total, 22 (3.4%) children had LC 28, including 14 (63.6%) males and 8 (36.4%) females. The median (IQR) age was 10.5 (IQR: 7.5–13.3) years. The majority had no basic diseases (68.2%) and had received two COVID-19 vaccine doses (90.9%). The median time between their last vaccination and infection was 13.0 (IQR: 11.8–14.2) months, with the majority (70.0%) having been vaccinated for longer than 12 months. The median number of symptoms during acute infection was 5.5 (IQR: 1.8–8.0), with most (59.1%) having more than three symptoms. Moreover, 40.9% of children felt that their symptoms were serious, with a subjective feeling score of more than 40 (33.0, IQR: 18.8–50.0). Most children (77.3%) were treated by home medications. The characteristics of the study population are listed in Table 3.

**Table 3:** Socio–demographic characteristics of LC 28 in school-aged children (*n* = 22)

Characteristic	Frequency	%
**Gender**		
Male	14	63.6
Female	8	36.4
**Age (years)**		
3–6	5	22.7
7–9	2	9.1
10–12	9	40.9
13–16	6	27.3
**Presence of basic diseases** ^*^		
No	15	68.2
Yes	7	31.8
**Vaccination doses**		
None/one	2	9.1
Two	20	90.9
**Time between last vaccination and COVID-19 infection (** ***n*** ** = 20)** ^**#**^		
<6 Months	1	5.0
6–12 Months	5	25.0
>12 Months	14	70.0
**Number of symptoms**		
0	1	4.5
1–3	8	36.4
>3	13	59.1
**Subjective feeling score**		
<20	7	31.8
20–40	6	27.3
>40	9	40.9
**Treatment**		
Self-medication at home	17	77.3
Required medical attention	2	9.1
Both self-medication and required medical attention	2	9.1
Did not take medication	1	4.5

^*^Basic diseases included allergic rhinitis (4, 18.2%), eczema (2, 9.1%), food allergies (3, 13.6%), and history of surgery (1, 4.5%). ^#^Missing data

**Fig. 4:**
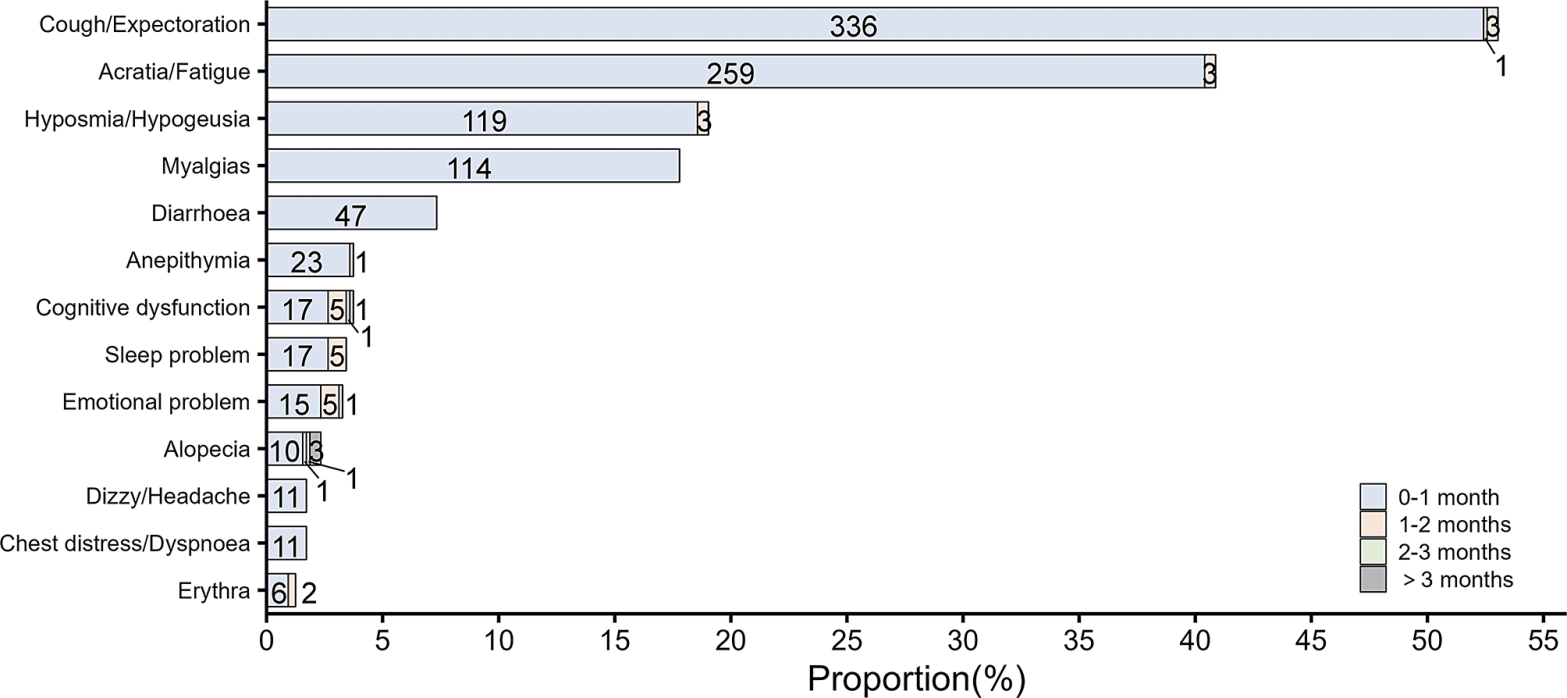
Prevalence and duration of LC 28 in school-aged children with COVID-19 infection

In terms of specific symptoms, the prevalence of LC 28 from the highest to the lowest was: alopecia (33.3%, 5/15), cognitive dysfunction (29.2%, 7/24), emotional problems (28.6%, 6/21), erythra (25.0%, 2/8), sleep problems (22.7%, 5/22), anepithymia (4.2%, 1/24), hypoosmia/hypogeusia (2.5%, 3/122), cough/expectoration (1.2%, 4/340), acratia/fatigue (1.1%, 3/262), myalgias (0.0%, 0/114), diarrhea (0.0%, 0/47), dizzy/headache (0.0%, 0/11), and chest distress/dyspnea (0.0%, 0/11). In terms of time distribution, the number of symptoms within 28 days was 4.0 (IQR: 2.0–6.0) and the number of symptoms that lasted for longer than 28 days was 1.0 (IQR: 1.0–2.0). The detailed results are shown in Fig. 4.

## Discussion

On December 7, 2022, the Chinese government announced 10 measures to end the zero COVID policy, which was in effect for longer than 2 years [12]. Most of the stringent preventive measures, such as mandatory PCR testing, are no longer required. However, the ease of restrictions has contributed to the emergence of new outbreaks predominantly by the SARS-CoV-2 omicron lineages BA5.2 and BF.7 in many cities [13]. A study from Guangzhou included 1,500 patients who attended hospitals during January 5–14, 2023, and found a positive rate of 61.5%, with 66.2% of patients aged 1–17 years. The study model predicted an infection rate of 80.7% for the entire population and 85.1% for individuals aged 1–17 years at 1 month after the end of the zero COVID policy [11]. In our study, the COVID-19 positive rate of children aged 3–16 years was 68.5%, which is comparable to the positivity rate in the same age group in Guangzhou during the same period. In total, 176 children were unaware of their COVID-19 infection; therefore, asymptomatic infections among the study population could not be ruled out. As a result, the COVID-19 positivity rate may be higher than 68.5%. On the one hand, the higher positivity rate may be related to the fact that most children received their last dose of vaccine more than 1 year ago. On the other hand, considering that the Omicron variant affects the lower respiratory tract rather than the upper respiratory tract, children with more immature and relatively smaller upper airways than adults are more susceptible [14].

In the present study, children had fewer and milder symptoms during the acute infection and used self-medication at home, which differed significantly from the trends in adults [15]. Fever, cough/expectoration, and rhinobyon were the most common symptoms, but most symptoms lasted for only 1–3 days, with a small number of symptoms lasting for 4–7 days. These results are in line with those of other studies [16, 17]. It is suggested that parents should not be excessively worried if their children develop fever or other symptoms of COVID-19 infection. Self-medication at home and observation can be advised for such patients, with the advice to seek medical treatment after 3 days in case of no improvement, to avoid crowding of medical resources.

In our study, most children recovered within 7 days from the top 10 acute symptoms of COVID-19. In particular, for fever, diarrhea, rhinobyon, and runny nose, more than 97.5% of patients were able to recover in a short time. However, more than 10% of children still had symptoms of cough/expectoration and hyposmia/hypogeusia after 7 days, which should be closely monitored by parents to prevent their children from developing long COVID-19.

Our scale showed a median score of 15.0 (IQR: 11.0–24.5) on a subjective feeling scale (total score: 100), suggesting that most children felt well during COVID-19 infection. The more unpleasant symptoms were fever, cough/expectoration, and rhinobyon, which are consistent with other studies [18]. Our multivariable regression model showed that, in contrast to junior children without basic diseases, senior children (aged 13–16 years) who had basic diseases, such as genetic disorders, birth defects, tumors, diabetes, hypertension, hepatitis, pneumonia, asthma, alle rgic rhinitis, eczema, food allergies, and history of surgery, had more severe subjective feelings. The presence of one or more basic diseases is associated with higher odds of developing moderate or severe/critical illness. Senior children are also at higher risks of experiencing COVID-19 symptoms, having a longer duration of illness, and having worse symptoms. These factors can influence the subjective feelings of illness [19, 20].

In the case of long COVID-19, the prevalence of LC 28 was only 3.4%, which is much lower than that in adults. This may be related to the faster recovery in children [21, 22]. Alopecia, cognitive dysfunction, emotional problems, erythrina, and sleep problems were common symptoms of LC 28 in children. As reported by Kathirvel et al. [23], Peluso et al. [24], and Cysique et al. [25], cognitive dysfunction, commonly known as “brain fog,” is a significant neuropsychological condition in LC, which particularly affects the areas of attention, memory, and executive functioning [26]. Mazza et al. [27, 28] found that neuropsychiatric symptoms, such as depression, anxiety, and insomnia, are associated with immunoinflammatory dysfunction and that abnormal immunoinflammatory responses play a non-negligible role in the generation, development, and worsening of neuropsychiatric symptoms. Furthermore, negative emotions (concerns about health, school, and medical care) may also contribute directly to the onset of emotional problems. In addition, children with LC 28 have a greater severity of symptoms and uncomfortable feelings in the acute phase. Parents are advised to pay more attention to children with worse symptoms, especially mental and psychological problems, after COVID-19, and to seek medical treatment if necessary.

### Limitations

This study had certain limitations. First, this study was conducted 2 months after the peak of COVID-19 infections, which may have led to a recall bias. Second, information about the children was provided by the parents, which may have led to information bias, although this approach has shown reliable in the routine Student Health Surveillance in Shenzhen. Third, a positive COVID-19 infection was not confirmed by a physician, which may have led to an erroneous diagnosis. Fourth, due to the limited number of schools and sample size, there may be selection bias. Nevertheless, this study fills a gap in the data on children infected with the SARS-CoV-2 omicron, acute/persistent symptoms, and symptom duration after the new COVID-19 policy in China, as well as provides a basis for the medical care of children, highlights the issues that parents need to focus on, and suggests the rationalization of medical resource allocation by the government.

## Conclusions

In the first wave of the COVID-19 epidemic after the policy change, there was a positivity rate of 68.5% in school-aged children in Shenzhen, China. In this wave of the epidemic, children had a short duration of acute symptoms and less subjective feelings, so they can self-medicate at home, which can minimize medical crowding. Children with basic diseases should be given attention and prompt medical attention when necessary. The prevalence of LC 28 is low in children, but parents should not ignore the mental or psychological problems of children after COVID-19 recovery.

## Data Availability

The datasets used and analyzed during the current study are not publicly available for ethical and privacy reasons but are available from the corresponding author upon reasonable request.
